# NAC, Tiron and Trolox Impair Survival of Cell Cultures Containing Glioblastoma Tumorigenic Initiating Cells by Inhibition of Cell Cycle Progression

**DOI:** 10.1371/journal.pone.0090085

**Published:** 2014-02-28

**Authors:** Massimiliano Monticone, Razieh Taherian, Sara Stigliani, Elisa Carra, Stefano Monteghirfo, Luca Longo, Antonio Daga, Mariella Dono, Simona Zupo, Walter Giaretti, Patrizio Castagnola

**Affiliations:** IRCCS AOU San Martino – IST, Genova, Italy; University of Florida, United States of America

## Abstract

Reactive oxygen species (ROS) are metabolism by-products that may act as signaling molecules to sustain tumor growth. Antioxidants have been used to impair cancer cell survival. Our goal was to determine the mechanisms involved in the response to antioxidants of a human cell culture (PT4) containing glioblastoma (GBM) tumorigenic initiating cells (TICs). ROS production in the absence or presence of N-acetyl-L-cysteine (NAC), tiron, and trolox was evaluated by flow cytometry (FCM). The effects of these antioxidants on cell survival and apoptosis were evaluated by 3-(4,5-Dimethylthiazol-2-yl)-2,5-diphenyltetrazolium bromide assay (MTT) and FCM. The biological processes modulated by these drugs were determined by oligonucleotide microarray gene expression profiling. Our results showed that NAC, tiron and trolox impaired PT4 cell survival, had minor effects on ROS levels and caused wide deregulation of cell cycle genes. Furthermore, tiron and trolox caused inhibition of cell survival in two additional cell cultures containing TICs, FO-1 and MM1, established from a melanoma and a mesothelioma patient, respectively. NAC, instead, impaired survival of the MM1 cells but not of the FO-1 cells. However, when used in combination, NAC enhanced the inhibitory effect of PLX4032 (BRAF V600E inhibitor) and Gefitinib (EGFR inhibitor), on FO-1 and PT4 cell survival. Collectively, NAC, tiron and trolox modulated gene expression and impaired the growth of cultures containing TICs primarily by inhibiting cell cycle progression.

## Introduction

Reactive oxygen species (ROS) are generated at several cellular compartments during normal cell metabolism [1]. Mitochondria are considered the main source of ROS, at least in mitochondria-rich cell types, and the superoxide anion is the most abundant form of ROS that they generate at several redox centers [2]. Other relevant sources of ROS are NAPDH oxidase, cytochrome P450 enzymes and xanthine oxidase; these are mainly located at the plasma membrane, endoplasmic reticulum and in the cytosol, respectively.

High levels of endogenous ROS may damage DNA, proteins and lipids, especially those in mitochondria which are closest to the main source of ROS, leading to cellular dysfunction and apoptosis [3]. A tight regulation of the intracellular redox status is therefore critical for cellular homeostasis and several enzymatic and non-enzymatic protective mechanisms have evolved to keep ROS levels in check [3]. Moderate levels of endogenous ROS instead, play a central role as second messenger molecules in the regulation of a number of critical cellular processes including cell survival [3], [4]. For instance, various reports have demonstrated that growth factors such as PDGF and EGF can stimulate ROS production. ROS, in turn, may directly or indirectly activate [5] several mitogen-activated protein kinases (MAPKs) [6], [7] or the nuclear factor of kappa light polypeptide gene enhancer in B-cells 1 (NF-kB) [8] and the AKT pathway [9]. Depending on the stimulus, these pathways are ultimately responsible to induce cell growth or apoptosis [5], [10]. Furthermore, several key proteins involved in transcription, signal transduction and in the execution of cell death or survival are directly regulated by ROS [3]. For other proteins, the redox regulation of their activity is indirect. For example, the administration of reductants was shown to suppress the dimerization and activation of the EGFR in rat pheochromocytoma cells PC12 but not in purified EGFR, indicating that this regulation is achieved through multiple intracellular processes [11]. The increase of intracellular ROS, induced by a variety of exogenous chemicals, may also impair cell proliferation by affecting cell cycle checkpoint functions mediated by the DNA damage response [12].

ROS levels in cancer cells were found to be higher than in normal cells [13]–[16]. Furthermore, persistent oxidative stress was observed in colorectal adenocarcinomas but not in adenomas [17]. A decrease of intracellular ROS via antioxidants administration was shown to impair proliferation or survival of several cell types, including colorectal adenocarcinomas, lymphomas and gliomas [18]–[20]. Several mechanisms were proposed to explain the antiproliferative effect displayed by antioxidants in cancer cells. The upregulation of P21waf, the inhibition of PKC, AKT and receptor tyrosine kinase (RTK) signaling, along with a decrease in NF-kB DNA binding activity, were shown to be elicited by the antioxidant N-acetyl-L-cysteine (NAC) in rat glioma cells [19]. In other cell models, such as lymphoma cells, alpha-tocopherol was shown to downregulate V-Myc Avian Myelocytomatosis Viral Oncogene Homolog (*MYC*), lactate dehydrogenase (*LDH*) and protein kinase C-alpha (*PRKCA*) gene expression [20].

Modulation of ROS levels is currently viewed as having therapeutic potential although the benefit of using antioxidants in both chemoprevention studies and in cancer therapy is still controversial [21]–[24]. Indeed some aggressive cancers often have multiple copies of genes belonging to the rat sarcoma viral oncogene homolog gene family (*RAS*) and *MYC* and display high levels of endogenous antioxidants [25]. Importantly, some antioxidants were shown to actually promote the development of prostate cancer in individuals without history of disease [26]. Such finding was recently confirmed in a prostate cancer mouse model by using NAC as antioxidant [27].

In this study we first established the effect of the administration of three different antioxidant drugs, NAC, trolox, and tiron on the survival of cell cultures containing glioblastoma (GBM) tumor initiating cells (TICs). Second, we studied the biological processes modulated by these drugs using flow cytometry (FCM) and gene expression profiling. The decision to use these three antioxidants was based on the fact that they have different structure and mechanisms of action. NAC is a thiol compound that acts both as a direct and indirect ROS scavenger since it is a precursor of reduced glutathione [28] and increases the activity of super oxide dismutase (SOD) [29]. Trolox (6-hydroxy-2,5,7,8-tetramethylchroman-2-Carboxylic Acid) is a vitamin E analog and a direct scavenger of peroxyl and alkoxyl radicals [30]. Tiron (1,2-dihydroxybenzene-3,5-disulfonate) is also a vitamin E analog and acts as a direct hydroxyl radical (•HO) and superoxide scavenger. Tiron is also a metal chelator [31].

Our hypothesis was that a true antioxidant effect would be translated into the modulation of genes involved in similar biological processes after exposure of the cells to any one of the three drugs. By contrast, non-antioxidant-related effects on gene expression and biological processes would be observed only after exposure of cancer cells to a specific drug. We used antioxidant concentrations sufficient to achieve a 50% reduction in cell survival in six consecutive days of exposure during which the cell culture medium containing the drug was changed every other day. This was done to reduce the possibility of observing tout-court toxic effects related to high drug concentration and, at the same time, to mimic more closely a chronic exposure to the drugs.

## Materials and Methods

### Cell cultures

Long-term cultures containing GBM TICs were obtained from surgical samples of tumors provided by the Neurosurgery Department of the San Martino Hospital in Genoa. An informed consent was obtained from all patients before surgery as required by the Ethic Board.

Patients and tumor characteristics from which cell lines used in this study were derived were recently described in detail by Monticone and coworkers [32], [34] as were cell isolation and culture methods [33]. Tumor initiating potential of the GBM long term cultures used in this study were reported in previous studies [33], [34].

Normal human astrocytes were purchased from ScienCell Research Laboratories (Carlsbad, CA) and cultured following the manufacturer's instructions.

The FO-1 melanoma cells with characteristics of cancer stem cells [36] were obtained from human melanoma [35] and were cultured as previously described [36].

The MM1 cells were obtained from a human malignant mesothelioma and displayed tumor initiating cell characteristics as previously reported [37].

### Genetic characterization of cells

The PT4 cells were positive for the vIII mutation in the extracellular domain of the EGFR (Eleonora Gambini, personal communication). By direct genomic sequencing PT2 and PT4 cells were found to be wild-type (wt) for the exon 18–21 sequences of the *EGFR* gene. In addition these cells were wt for: *PI3KCA* (exon 9 and 20), relevant for the GBM biology [38], [39]; *KRAS* (exon 2), relevant for cell-redox balance [40], [41] and *BRAF* (exon 15), downstream of *EGFR* in the EGF signaling pathway (data not shown). By nucleotide sequencing of the *TP53* cDNA, PT2 cells were found homozygotes for the single nucleotide variation (SNV) CCC>CGC at codon 72, resulting in a Pro>Arg non-pathogenic substitution, whereas PT4 cells were wt.

Multiplex ligase probe amplification (MLPA) of PT4 cells displayed amplification of the *EGFR* locus, gain at the MDM4 and TSC1 loci, loss at the *RET* and *CCND2* loci and biallelic loss at the *CDKN2A* and *CDKN2B* loci [32]. PT2 cells displayed gain at the *EGFR, MET, SMO, BRAF* loci; amplification of *MYC* and *MDM2* loci; loss at *RET, PTEN, CCND1* loci and biallelic loss at the *CDKN2A* and *CDKN2B* loci [32].

Direct genomic sequencing of MM1 cells showed wt sequences for the following genes: *EGFR* (exons 18–21), *PI3KCA* (exon 9 and 20), *KRAS* (exon 2) and *BRAF* (exon 15) (data not shown). The cDNA sequence of *TP53* in MM1 cells showed homozygosity for the SNV C/G at codon 72 resulting in a Pro>Arg non-pathogenic substitution.

FO-1 cells were wt for the following genes: *EGFR* (exons 18–21), *PI3KCA* (exon 9 and 20), and *KRAS* (exon 2) but carried the codon 600 GTG>GAG V>E mutation in the exon 15 of the *BRAF* gene (data not shown). The cDNA sequence of *TP53* in FO-1 cells showed that they were heterozygotes for both the SNV CCC>CGC at codon 72, resulting in a Pro>Arg non-pathogenic substitution, and for the SNV G/A resulting in a synonymous CGA>CGG change at codon 213.

### Chemicals

NAC, tiron, trolox and PLX4032 were obtained from Sigma-Aldrich (Schnelldorf, Germany). NAC and tiron were dissolved in H_2_O at 2 M concentration. Gefitinib (AstraZeneca, Basiglio, Italy) and Trolox were dissolved in ethanol. PLX4032 was dissolved in DMSO.

NAC and tiron control cultures were additioned with equal amounts of H_2_O, whereas trolox and Gefitinib control cultures were treated with equal amounts of ethanol.

To the PLX4032 control cultures were added equal amounts of DMSO (0.1% vol/vol final concentration).

### Cell survival

Cell survival was evaluated at different time intervals using the MTT colorimetric assay as recommended by the manufacturer [42].

### Fluorochromes and FCM analysis

To measure the DNA content in cell nuclei, each sample was stained with 4,6-diamidino-2-phenylindole-2-hydrocloride (DAPI) as described by Otto [43] using a CyflowML multiparameter flow cytometer (Partec).

Global intracellular ROS were determined by incubating the cells with 5-(and-6)-chloromethyl-2′,7′-dichlorodihydrofluorescein diacetate, acetyl ester (DCFDA). To assess superoxide anion generation specifically in mitochondria, cells were labeled with MitoSOX Red whereas mitochondrial mass was evaluated by incubating the cells with Mitotracker green. Tetramethylrhodamine, ethyl ester (TMRE) was used to evaluate the mitochondrial membrane potential, delta psi. All fluorescent dyes were from Life Technologies (Monza, Italy) and cellular staining was performed according to the manufacturer's instructions.

The analysis of the fraction of cells actively synthesizing DNA was performed according to the method published by Dolbeare et al. [44] employing a pulse of 90 min of 10 µM 5-bromo-2′-deoxyuridine (BrdU) (Sigma-Aldrich). Samples were subjected to FCM analysis using a CyAn ADP analyzer (Dako, Glostrup, Denmark).

### Gene Expression Profiling

After addition of spike-in controls, 200 ng of RNA was amplified and labeled using one-color microarray based gene expression protocol v 6.0; labeled cRNAs were hybridized to Human GE 4×44K v2 Microarray (Agilent Technologies, Santa Clara, CA). Slides were scanned by the Agilent G2565BA scanner and images were processed by Feature Extraction software v.10 (Agilent Technologies). The raw data were imported into GeneSpring 12.1 software (Agilent Technologies) and a quantile normalization was applied.

Microarray data have been deposited in the National Center for Biotechnology Information Gene Expression Omnibus and are accessible via: http://www.ncbi.nlm.nih.gov/geo/query/acc.cgi?token=xxsxlqouuaasmzk&acc=GSE44099.

### Pathways identification by DAVID Bioinformatics Resources

Differentially expressed genes were examined using the tools of the web-accessible DAVID 6.7 program (http://david.abcc.ncifcrf.gov/) [45], [46].

### qPCR analysis

Relative quantification of RNA transcripts was performed by real-time quantitative RT-PCR (qPCR) using the SYBR Green chemistry. The gene coding for the ribosomal protein L19 (RPL19) was used as the endogenous normalization control.

Specific primers for each transcript were designed across a common exon-exon splice junction by the Primer Express software (Applied Biosystems, Monza, Italy) (Table S1).

### SDS PAGE and immunoblot analysis

Cell lysates were prepared as described previously [47]. SDS PAGE and immunoblotting were performed using 4–12% polyacrylamide gels (Invitrogen) following manufacturer's instructions. The blotting membranes were from Millipore (Vimodrone, Italy). For the list of the antibodies used see Text S1. Horseradish peroxidase conjugated goat anti-rabbit or goat anti-mouse IgGs and the SuperSignal west Pico Chemiluminescent substrate (Thermo Scientific, Rockford, IL) were used for immunodetection. Chemiluminescent signals at 95% saturation levels were acquired by a UVITec gel documentation system (Cambridge, UK). Densitometric analysis was performed by the Alliance 4.7 software (UVItec).

## Results

### Cultures containing GBM TICs have an enhanced production of ROS

**Figure 1:**
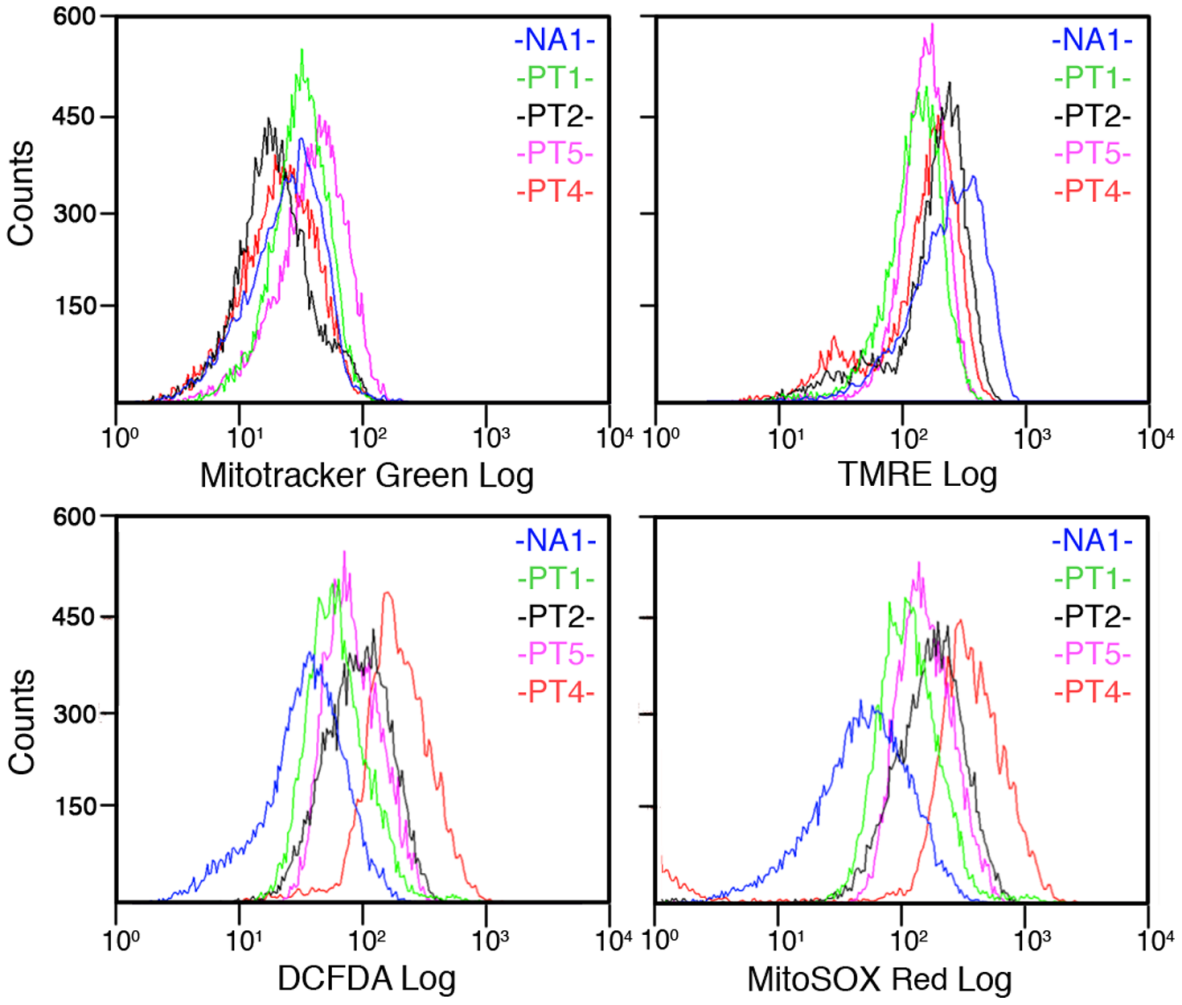
Cell cultures containing GBM TICs display higher endogenous ROS generation than normal astrocytes. Representative experiment of FCM analysis of normal human astrocytes (NA) and cell cultures containing GBM TICs obtained from four different patients (PT1, PT2, PT4, PT5) incubated with the indicated fluorescent probe. Mitotracker Green, TMRE, DCFDA and MitoSOX Red were used to evaluate mitochondrial mass, mitochondrial proton gradient, global ROS and mitochondrial superoxide, respectively.

To compare ROS production levels in cultures containing GBM TICs with normal human astrocytes (NA), four cultures obtained from four different GBM patients (see Materials and Methods) and a primary culture of NA (NA1) were incubated with DCFDA and MitoSOX Red. Fluorescent emission was measured by FCM analysis. All cultures containing GBM TICs showed an enhanced production of both global ROS and mitochondrial superoxide compared with NA1 as measured by DCFDA and MitoSOX Red, respectively (Figure 1). This difference was unrelated to mitochondrial mass because the signal emission generated by Mitotracker green, which is specifically incorporated in mitochondria, was in the same range of values for all cells tested (Figure 1). A possible inverse relationship between increased ROS production and decreased proton gradient across the inner mitochondrial membrane (delta psi) was suggested by the measure of signal emitted by the TMRE whose accumulation in the mitochondrial matrix is dependent from the delta psi. In fact, all cultures containing GBM TICs showed a decrease of TMRE-dependent fluorescence emission compared with NA1 (Figure 1).

Further experiments showed that PT2 and PT4 cells (those that displayed the highest ROS generation ability among cultures containing GBM TICs) generated more global ROS compared to a second lot of NA (NA2), whereas a lower generation of mitochondrial superoxide was observed in both PT2 and PT4 (Figure S1). However, the NA2 culture showed a higher mitochondrial mass compared to both PT2 and PT4, which may explain the higher fluorescent signals obtained with both MitoSOX Red and TMRE in NA2 (Figure S1).

### NAC, tiron and trolox impair survival of cultures containing GBM TICs

The increased production of global ROS in PT2 and PT4 cultures containing GBM TICs compared with two different lots of NA suggested that either ROS might play a permissive role with respect to the proliferation ability of these cultures or, conversely, the cultures containing GBM TICs might be stressed by the ROS production. To test these hypotheses, the PT4 culture was challenged with antioxidant drugs and cell survival was evaluated. We choose the PT4 cell culture because it showed the highest constitutive ROS levels and this could make it the best candidate to test the effects of antioxidants.

**Figure 2:**
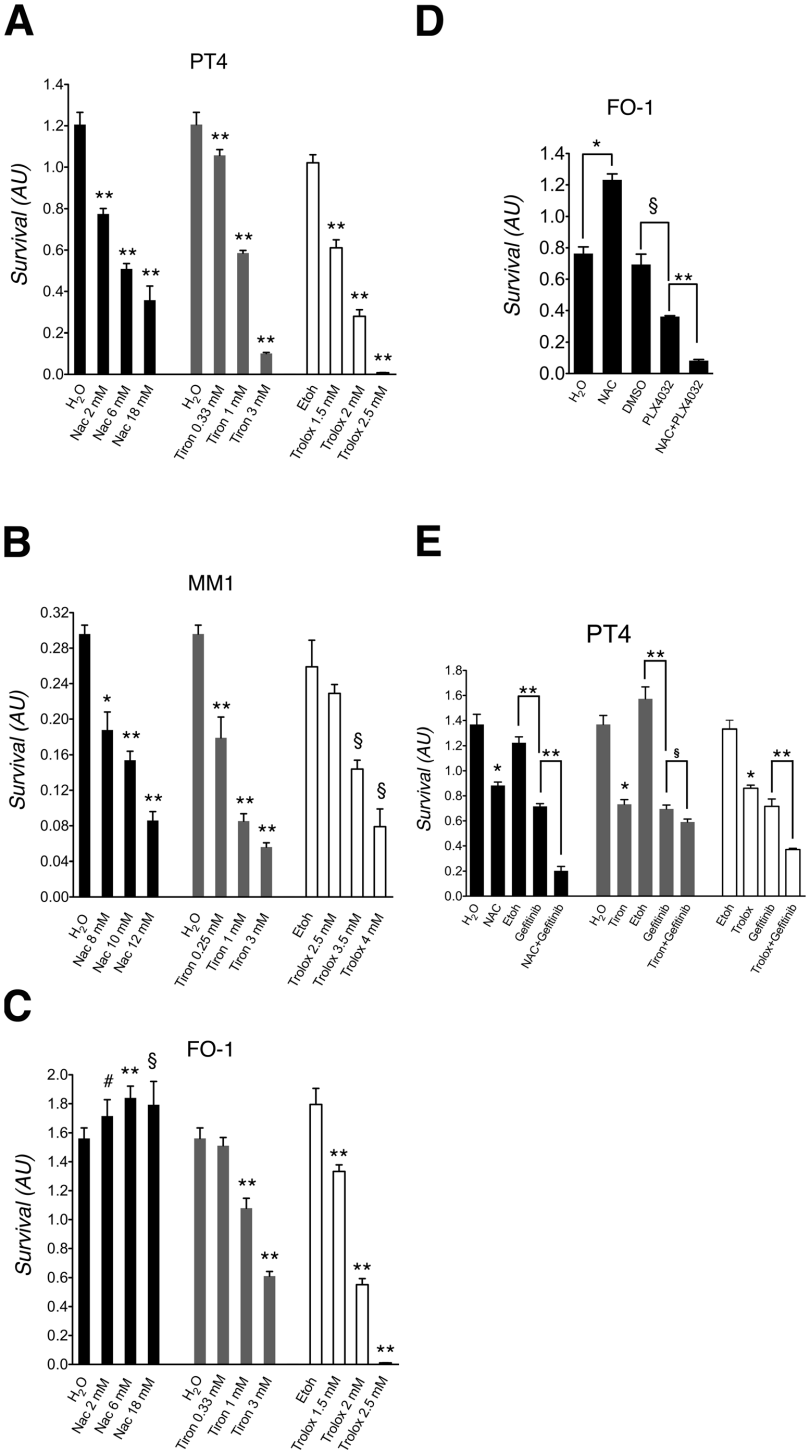
Cell survival of the PT4, MM1 and FO-1 cell cultures containing TICs. Survival of PT4 cells (A, E); MM1 cells (B) and FO-1 cells (C, D) after 6 days of treatment with the indicated substances and solvent controls. The medium renewal schedule was identical to that used for the cultures containing GBM TICs (see Introduction). Cell survival is expressed in arbitrary units as evaluated by MTT analysis. Standard deviations are indicated as vertical bars (n = 3 independent assays). DMSO concentration in (D) was 0.1% vol/vol. Drug concentrations in (D) were: NAC 20 mM, PLX4032 10 µM. Gefitinib final concentration in (E) was 3.9 µM. #The unpaired t-test was significant at P<0.05. §The unpaired t-test was significant at P = 0.01 or less. *The unpaired t-test was significant at P<0.001. **The unpaired t-test was significant at P<0.0001.

As antioxidants we used NAC, tiron and trolox; these compounds have different chemical groups able to inactivate ROS (see Introduction). As control conditions throughout the study, we added an equal amount of H_2_O (the solvent used for NAC and tiron) or of ethanol (the solvent used for trolox) to the media. The extent of cell survival was determined by MTT analysis. This experiment showed that the three drugs inhibited cell survival in a dose dependent manner (Figure 2A). We extrapolated IC50 doses from dose-response curves (shown in Figure 2) and verified experimentally (not shown) that after 6 days of exposure with 3 mM, 0.7 mM and 1.8 mM of NAC, tiron and trolox respectively, the PT4 cell survival was about 50% compared to control cultures. Unless otherwise stated, these doses were used for all subsequent experiments with PT4 cells. Evaluation of treated and untreated PT4 cells by phase contrast microscopy confirmed the MTT data (Figure S2).

Further incubation of PT4 cells (up to 10 days of total treatment time) with the lowest two concentrations of the three drugs tested in the dose-response curve, showed a significant decrease in cell survival compared to control cultures except for the lowest tiron concentration (Figure S3).

A dose-dependent decrease in cell survival induced by NAC, tiron and trolox was also observed in PT2 cultures containing GBM TICs (Figure S4).

The reduction of PT4 cell survival caused by antioxidants could be due to enhanced apoptosis or necrosis. Therefore we used APC-conjugated annexin V, C12-resazurine and Sytox blue to discriminate early apoptotic, metabolically active cells, late apoptotic, and necrotic cells by FCM. After 6 days of culture in presence of all three drugs, we detected only a modest increase of the early and late apoptotic cells compared to control cultures (data not shown).

### NAC, tiron and trolox affect the cell cycle of cell culture containing GBM TICs

**Figure 3:**
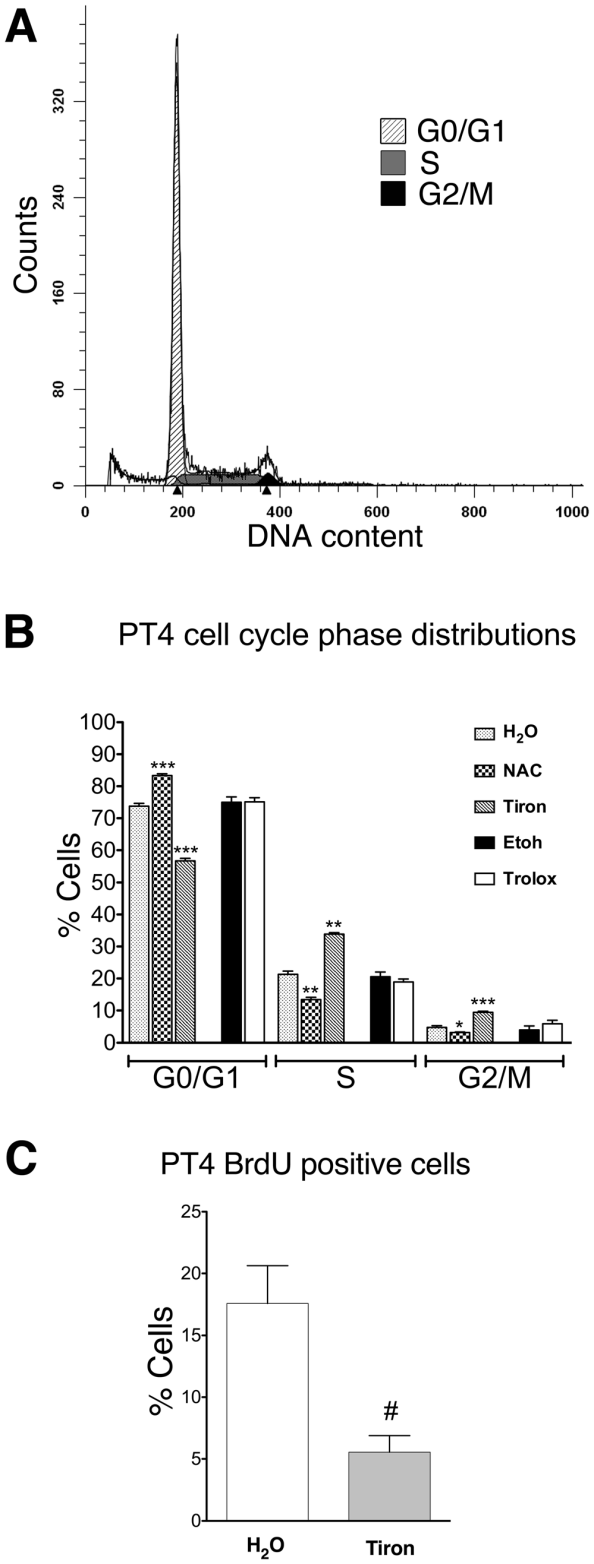
NAC and tiron modify the distribution of the cell cycle phases in the PT4 cell culture containing GBM TICs. Representative experiment of high resolution FCM analysis of DAPI-stained nuclei of the H_2_O control PT4 culture containing GBM TICs after 48 h of exposure (A). Analysis of the percentage of PT4 cells in the cell cycle phases as determined by the ModFit LT™ software after 48 h of exposure with the indicated substances at the IC50 concentration and solvent controls (B). Percentage of PT4 cells in S phase actively synthesizing DNA after 48 h of exposure to tiron at the IC50 concentration with respect to control condition (H_2_O) performed by BrdU labeling and FCM analysis (C). Standard deviations are indicated as vertical bars (n = 4 independent assays, B ; n = 3 independent assays, C). #The unpaired t-test was significant at P<0.05. *The unpaired t-test was significant at P<0.001. **The unpaired t-test was significant at P<0.0001. ***The unpaired t-test was significant at P<0.00001.

To establish whether the antioxidant treatment affected the distribution of the cell cycle phases in the PT4 culture, we analyzed the nuclear DNA content by high resolution-DNA FCM (hr-DNA FCM) using DAPI staining (Figure 3). This analysis showed that a 48 h treatment with NAC increased the percentage of PT4 cells in the G0/G1 phase (with a concurrent reduction of cells in both S and G2/M phases) compared to control cultures (Figure 3). The treatment with tiron, instead, increased the percentage of PT4 cells in both the S and G2/M phases (along with a reduction of cells in the G0/G1 phase) compared to control cells (Figure 3). Furthermore, the treatment of PT4 cells with trolox did not induce changes in the cell cycle phases compared to the control condition (Figure 3).

The same hr-DNA FCM analysis was also performed with PT2 cells and confirmed the increased percentage of cells in the G0/G1 phase and the reduction of those in the G2/M phases after NAC treatment (Figure S5). Similarly, the tiron-induced increase of cells in the S phase was confirmed although in the PT2 cells this was at the expense of the G2/M phases and not G0/G1 phase (Figure S5). It is worth noting that in the PT2 culture trolox induced a modification of the distribution of cells in the cell cycle phases (Figure S5). Specifically, an increase of the percentage of cells in the G0/G1 phase along with a reduction of cells in the S phase was observed (Figure S5).

To better investigate how tiron affected the S phase in PT4 cells, we performed BrdU incorporation followed by immunodetection and FCM analysis. A lower percentage of BrdU-positive cells after tiron treatment compared to control cells was observed (Figure 3). The same result was obtained with PT2 cells (figure S5).

### Antioxidants differently affect the intracellular ROS levels and the phosphorylation status of AKT, ERK1/2 and NF-kB in the PT4 culture containing GBM TICs

**Figure 4:**
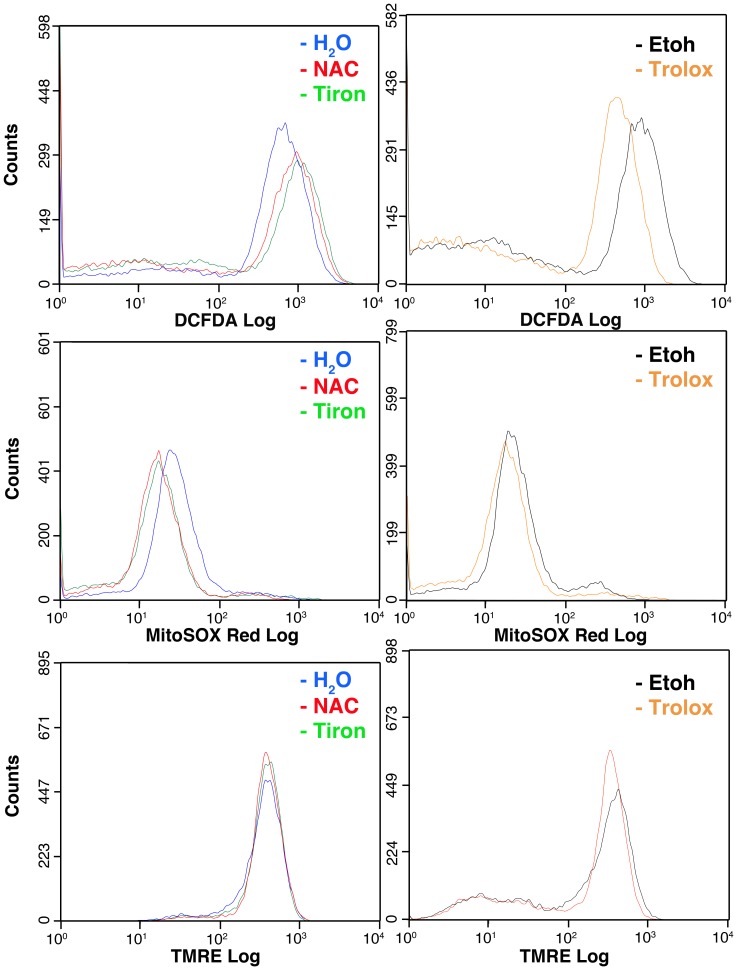
NAC, tiron and trolox determine only modest changes in the ROS levels in the PT4 cell culture containing GBM TICs. Representative experiment of FCM analysis of PT4 culture containing GBM TICs cells incubated with the indicated fluorescent probe after 48(containing NAC, tiron or trolox) replacement. DCFDA, MitoSOX Red and TMRE were used to evaluate global ROS, mitochondrial superoxide and mitochondrial proton gradient, respectively. This analysis showed that trolox reduced global cellular ROS levels but did not substantially modify mitochondrial superoxide levels. NAC and tiron, instead, slightly decreased mitochondrial superoxide levels while slightly enhancing global cellular ROS levels. This analysis also showed that the drugs used in this study did not induce changes of the mitochondrial proton gradient displayed by the PT4 cells in control conditions.

To evaluate whether the antioxidants used in this study were indeed able to reduce intracellular ROS levels, PT4 cells were preincubated with NAC, tiron, trolox or solvents for 48 h. Subsequently, the cells were transferred into suspension and incubated with DCFDA, MitoSOX Red and TMRE in the presence of NAC, tiron, trolox or solvents. All three antioxidants were able to slightly reduce the MitoSOX Red-dependent fluorescence emission compared to solvent treated cells. While the reduction of the DCFDA-dependent fluorescence was observed only for trolox, NAC and tiron slightly increased this fluorescence emission (Figure 4). The three antioxidants did not cause major changes of the mitochondrial delta psi as evaluated by TMRE fluorescence (Figure 4). Similar results were obtained with PT2 cells with the exception that tiron slightly increased MitoSOX Red fluorescence (Figure S6).

To assess whether NAC, tiron and trolox similarly affected the abundance and the phosphorylation status of AKT, ERK1/2 and NF-kB, we performed immunoblot analyses of PT4 cell lysates after the cells were treated for 48 h. These analyses showed that each antioxidant drug affected the amount and the phosphorylation status of those proteins in a specific pattern (Figure S7). In particular, densitometric analysis (not shown) indicated that NAC induced a reduction of the pAKT/AKT ratio and an increase of the pERK1/2/ERK1/2 and pNF-kB/NF-kB ratios compared to controls (H_2_O); tiron displayed an increase of the pAKT/AKT, pERK1/2/ERK1/2 and pNF-kB/NF-kB ratios compared to controls while trolox showed an increase of the pAKT/AKT and the pNF-kB/NF-kB ratios and a reduction of the pERK1/2/ERK1/2 ratio compared to controls (ethanol).

In conclusion, the inhibition of the PT4 cell survival displayed by the three antioxidant drugs could not be explained with a consistent pattern of phosphorylation changes of the three investigated protein kinases. Therefore we adopted a gene expression profile approach to address this issue.

### NAC and trolox affect cell survival of the PT4 culture containing GBM TICs by mainly inhibiting cell cycle genes whereas tiron induces hypoxia genes

To investigate the molecular basis underlining the effect of NAC, trolox and tiron on PT4 cells, we determined their gene expression profiles using oligonucleotide microarrays after 48 h and 6 days of drug exposure. Raw data were normalized, filtered and then analyzed by calculating the ratio between experimental conditions and corresponding solvent control expression levels. We chose an arbitrary 2.0 fold change cutoff to consider a given probe set as differentially regulated.

To gain a mechanistic understanding of the processes affected by the tested drugs, the DAVID tools [45], [46] were used to identify Gene Ontology (GO) biological processes that were significantly over-represented. Statistically significant GO biological processes associated with NAC, trolox and tiron regulated genes were identified and the three top ranking ones are listed in Table 1. At 48 h both NAC and trolox showed a deregulated expression of genes involved in the cell cycle whereas tiron affected genes related to oxygen level response, glycolysis and angiogenesis. Specifically, tiron upregulated all sixteen genes belonging to the GO biological process response to oxygen level such as *CA9* and *TRFC*, and all eleven genes belonging to the angiogenesis GO biological process such as *VEGFA* and *ANG* (data not shown). In addition, after 48 h of exposure, NAC also deregulated genes affecting the mitotic cell cycle and transcription biological processes whereas trolox modulated the expression of genes implicated in the M phase of mitotic cell cycle and in DNA replication. After 6 days of exposure we observed that: NAC deregulated genes involved in response to hypoxia, cell adhesion and system process; trolox modulated the expression of genes implicated in the M phase of the mitotic cell cycle, microtubule-based and cytoskeleton organization; tiron caused a deregulation of cell cycle, M phase and DNA replication. A full list of the deregulated genes by NAC, trolox and tiron in PT4 cells included in each GO biological processes listed in Table 1 is provided in the Table S2.

**Table 1:** Top three gene ontology biological processes resulting from the analysis of differential regulated genes between antioxidant drugs and the respective solvent controls.

Tiron vs. H_2_O control 48 h exposure				Tiron vs. H_2_O control 6 d exposure			
Gene ontology biological processes	Count	%	P-value	Gene ontology biological processes	Count	%	P-Value
response to oxygen levels	16	5.6	1.3E-08	cell cycle	178	13.1	1.4E-42
glycolysis	7	2.4	9.1E-05	M phase	104	7.7	4.7E-37
angiogenesis	11	3.8	1.7E-04	DNA replication	59	4.4	3.4E-21

Trolox vs. etoh control 48 h exposure				Trolox vs. etoh control 6 d exposure			
Gene ontology biological processes	Count	%	P-value	Gene ontology biological processes	Count	%	P-value
cell cycle	244	6.8	4.4E-17	M phase of mitotic cell cycle	37	3.3	4.7E-08
M phase of mitotic cell cycle	96	2.7	6.4E-17	microtubule-based process	37	3.3	4.7E-07
DNA replication	83	2.3	6.8E-15	cytoskeleton organization	52	4.6	2.0E-06

NAC vs. H_2_O control 48 h exposure				NAC vs. H_2_O control 6 d exposure			
Gene ontology biological processes	Count	%	P-value	Gene ontology biological processes	Count	%	P-value
cell cycle	171	7.3	3.4E-15	response to hypoxia	7	7.4	7.6E-05
mitotic cell cycle	101	4.3	7.4E-15	cell adhesion	13	13.7	2.1E-04
transcription	344	14.8	1.0E-11	regulation of system process	8	8.4	1.2E-03

This analysis was performed using the DAVID program (see MM). Count indicates the number of genes belonging to the indicated gene ontology biological processes that we found regulated by a factor 2; % indicates the percentage of regulated genes belonging to the indicated biological process compared to the total number of regulated genes and included in the DAVID database; P-value indicates the probability that the indicated biological process would result by random selection.

Among the three antioxidants, only trolox showed deregulation of genes related to the oxidation reduction GO biological processes (at 6 days of treatment) but the associated P-value 3.1E-2 was four orders of magnitude lower compared to that associated to the third top ranking GO biological process modulated by trolox at the same time point (data not shown).

An analysis of the total number of deregulated genes by each drug at each time point showed that NAC and trolox had an earlier impact on gene expression compared to tiron and that only a very small number of genes were deregulated by all three drugs (Figure S8).

### Validation at the transcript and protein levels of the differential gene expression by NAC, tiron and trolox

**Figure 5:**
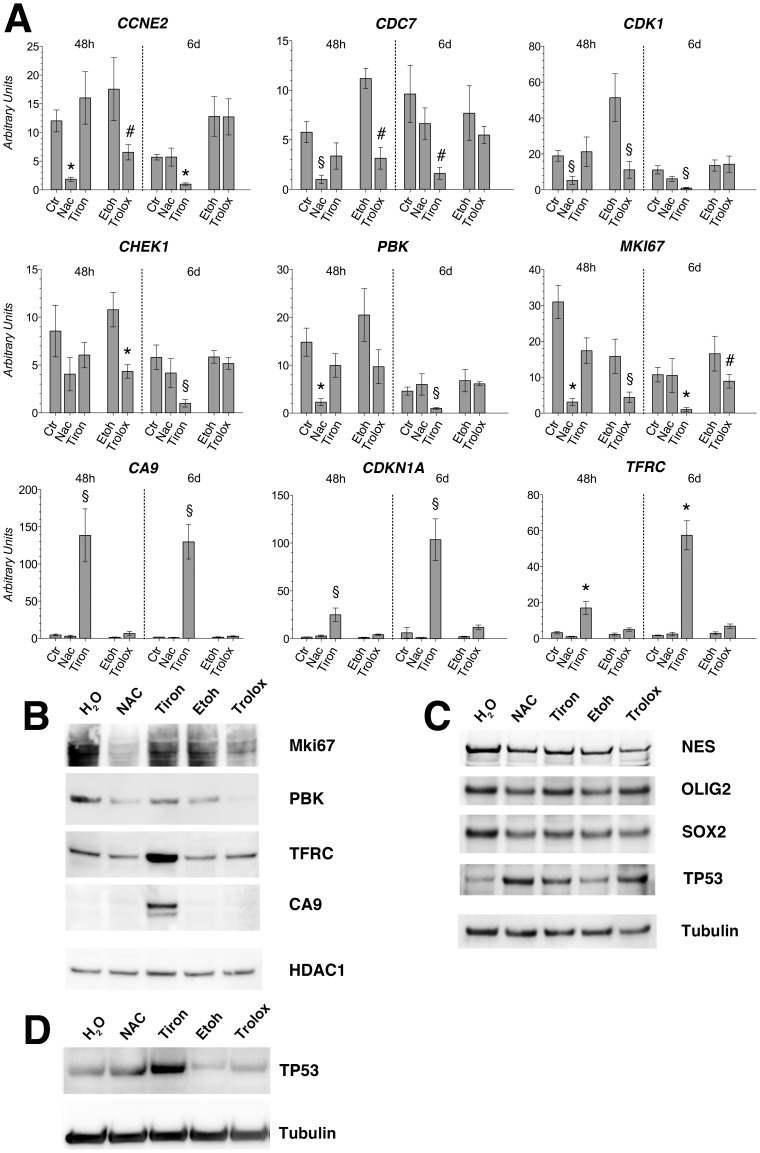
Validation of gene expression regulation by Real-time RT-PCR and Immunoblot analysis. (A) Real-Time RT-PCR analysis performed with RNA extracted from the PT4 cell culture containing GBM TICs to validate the microarray data. This was accomplished on randomly selected genes from Table S3 and showed, in arbitrary units. Expression levels are relative to the expression of the housekeeping gene transcript coding for the ribosomal protein L19 (RPL19). Standard deviations are indicated as vertical bars (n = 3 independent assays). Gene name symbols are those approved by the Human Genome Organization Gene Nomenclature Committee (http://www.genenames.org/). Standard deviations are indicated as vertical bars (n = 3 independent assays). #The unpaired t-test was significant at P<0.05. §The unpaired t-test was significant at P<0.01. *The unpaired t-test was significant at P<0.001. (B) Western blot analyses were performed with lysates of the PT4 cell culture containing GBM TICs treated for 48 h with the indicated antioxidant drug and challenged with anti MKi67, Pdz-binding kinase (PBK), transferrin receptor (TFRC), carbonic anhydrase 9 (CA9). (C) Western blot analyses were performed with lysates of the PT4 cell culture containing GBM TICs treated for 6 days with the indicated antioxidant drug and challenged with anti nestin (NES), oligodendrocyte transcription factor 2 (OLIG2), SRY (sex determining region Y)-box 2 (SOX2) and tumor protein 53 (TP53) antibodies. (D) Western blot analyses were performed with lysates of the PT4 cell culture containing GBM TICs treated for 48 h with the indicated antioxidant drug and challenged with anti tumor protein 53 (TP53) mAb. (B–D) Immunoblotted membranes were subjected to multiple antibody challenging, stripping, control of effective stripping, and re-challenging with a different antibody. The last antibody used was an anti histone deacetylase 1 (HDAC1) (B) or an anti tubulin alpha (D) to show equal loading.

To validate the observed gene expression changes, we performed qPCR analyses of arbitrarily selected gene transcripts involved in the control of cell cycle (*CCNE2, CDC7, CDK1, CHEK1, MKI67 and PBK*) and response to hypoxia (*TFRC, CDKN1A* and *CA9*) that were found deregulated by NAC, tiron and trolox. The results obtained were consistent with the microarray data (Figure 5A).

To validate the mRNA data at the protein level, we analyzed the expression of two key proteins involved in cell proliferation, Mki67 and PBK, and in the hypoxia response, TFRC and CA9, by immunoblot analysis of PT4 cell lysates. As expected, Mki67 and PBK protein levels were reduced in NAC and trolox treated cells whereas TFRC and CA9 were dramatically increased in tiron treated cells compared to solvent controls after 48 h of exposure (Figure 5B). Similar results were obtained for PBK and CA9 after 6 days of exposure whereas TFRC was upregulated also in trolox treated cells in addition to tiron (Figure S9). Ki67 was reduced in both NAC and tiron treated cells but it did not change significantly compared to control cultures in trolox treated cells (Figure S9).

### NAC, tiron and trolox do not affect the expression of the stemness-related genes *NEST*, *OLIG2* and *SOX2*, of the PT4 culture containing GBM TICs

To assess whether the antioxidants used in this study affected the stemness of PT4 cells, we investigated the expression levels of three stemness-related markers of GBM TICs: Nestin, OLIG2 and SOX2. Overall, the immunoblot analysis showed no major changes in these three proteins after 6 days of exposure to NAC, tiron and trolox (Figure 5C), as expected from the gene expression profile analysis (Table S3).

A potential growth inhibition due to differentiation of the GBM PT4 cells induced by the three drugs was also excluded since no changes in neuronal, glial and oligodendrocyte differentiation markers were detected after 6 days of treatment. The expression values for these central nervous system transcripts in each experimental condition are shown in Table S3.

### NAC, tiron and trolox treatments of PT4 cultures containing GBM TICs induce upregulation of the tumor protein 53 (TP53) and inhibit the phosphorylation of the retinoblastoma (RB) protein

TP53 is known to induce growth arrest and apoptosis and is mainly regulated at the protein level. To investigate whether TP53 expression was affected by the antioxidant treatment in PT4 cells, we performed an immunoblot analysis at 48 h and 6 days of exposure. TP53 was upregulated in all experimental conditions compared to controls (Figure 5C and 5D). In details, TP53 was slightly upregulated by NAC and trolox after 48 h of treatment (Figure 5C) and then distinctly upregulated by these drugs after 6 days (Figure 5D) compared to controls (H_2_O and ethanol, respectively). Tiron instead, showed a strong upregulation already at 48 h of treatment compared to control (Figure 5D). This tiron-induced upregulation of TP53 was still evident, although to a lesser extent, after 6 days of exposure (Figure 5C).

**Figure 6:**
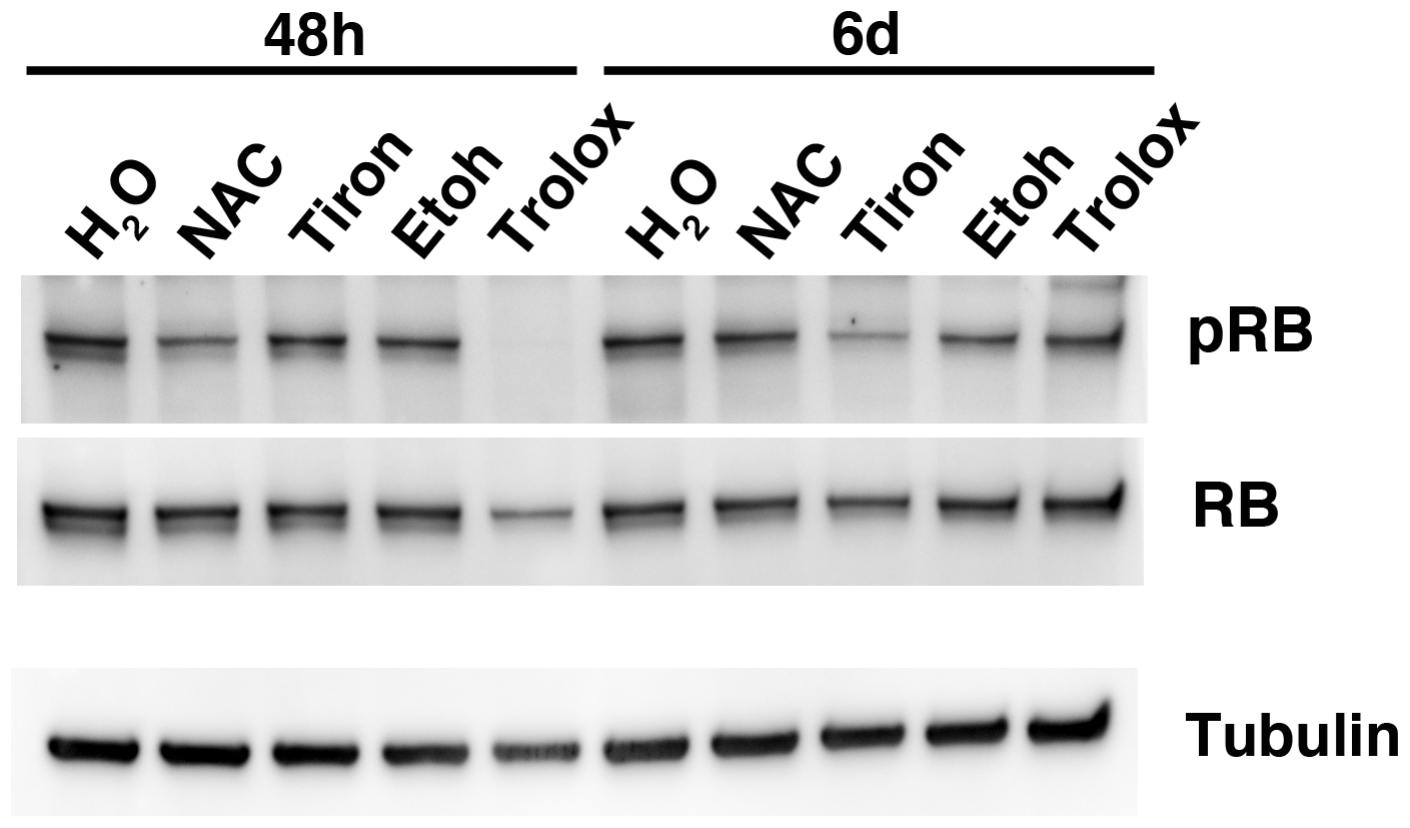
NAC, tiron and trolox treatments of the PT4 cultures containing GBM TICs inhibit the phosphorylation of the retinoblastoma (RB) protein. Western blot analyses were performed with lysates of the PT4 cell culture containing GBM TICs treated for 48 h and 6 days with the indicated antioxidant drug. The membrane was challenged first with an anti phosphorylated RB (Ser 807/811) antibody, then with an anti total RB antibody and lastly with an anti tubulin antibody. Stripping and control of effective stripping were performed before re-challenging of the membrane.

The phosphorylation status of the retinoblastoma protein (RB) plays a key role in blocking the progression through the cell cycle. Therefore we investigated if the three drugs used in our study affected this post translational modification in PT4 cells. Phosphorylated and total RB protein were detected in immunoblots using a densitometric analysis (Figure 6). At 48 h both NAC and trolox displayed a higher ratio of the active (hypophosphorylated) versus the inactive (phosphorylated) form of RB compared to controls (not shown). However, after 6 days of exposure to the same drugs no major changes in this ratio were detected (not shown). It should be noted though that the inactive (phosphorylated) RB was barely detectable in the trolox treated PT4 cells at 48 h (Figure 6). An increase of the ratio of the active over the inactive RB form with tiron was observed only at the 6 days time point (not shown).

### Effects of NAC, tiron and trolox on survival of cultures containing TICs from mesothelioma and melanoma

Then we asked if NAC, tiron and trolox have similar effects on survival of cultures containing TICs originated from other tumor types. To do this, we treated MM1 and FO-1 cultures that were previously obtained from mesothelioma and melanoma, respectively (see Materials and Methods). The treatment schedule was identical to that used for the PT4 culture (see Introduction). The MTT analysis showed a dose-dependent, reduced survival of MM1 cells after treatment with the three drugs (Figure 2B). Similarly, FO-1 cells showed a dose-dependent, reduced survival after treatment with tiron and trolox (Figure 2C). NAC, instead, did not induce major changes in FO-1 cell survival up to the 18 mM dose (Figure 2C). Longer incubation of MM1 cells, for up to 10 days, with the lowest and the intermediate concentrations (tested in the dose-response curve of the three drugs) showed a decreased cell survival compared to control cultures with 10 mM NAC, 0.25 mM and 1 mM tiron and with 3.5 mM trolox (Figure S3). The same experiment performed with FO-1 cells showed an effective reduction of cell survival with tiron concentrations of 0.33 mM and 1 mM; however this was not observed with NAC or trolox (Figure S3).

Interestingly, we determined that FO-1 cells had the common melanoma associated BRAF codon 600 V>E mutation (see MM), which renders BRAF constitutively active, whereas PT4 and MM1 cells were wild type in this same region. Hence we hypothesized that the constitutive activation of the EGFR/RAS/BRAF pathway could be responsible for the lack of sensitivity of FO-1 cells to NAC and therefore treated them with both NAC and PLX4032, a specific inhibitor of BRAF V600E. We used NAC at the concentration of 20 mM, which actually improved FO-1 cell survival, and PLX4032 at 10 µM. This concentration of PLX4032 yielded about 50% of cell survival when used individually. This co-treatment experiment showed that NAC further inhibited FO-1 cell survival induced by PLX4032 (Figure 2D).

These data prompted us to test whether a similar potentiation effect could be observed in PT4 cells, which tested positive for the *EGFR* vIII mutation, when treated with a combination of NAC or tiron or trolox and gefitinib, an EGFR inhibitor. These experiments, performed at doses yielding about 50% of cell survival when each drug was used individually, showed statistically significant enhancement of cell survival inhibition (Figure 2E). The potentiation effect was very minor, moderate or strong when tiron, trolox or NAC, respectively, was added together with gefitinib (Figure 2E).

## Discussion

This study addressed for the first time the effects of the antioxidant drugs, NAC, tiron and trolox in a GBM cell culture containing TICs. A significant impairment in cell survival was observed, presumably by mechanism(s) involving inhibition of cell cycle progression via different biological processes.

Our data demonstrated that among four GBM cell lines, PT4 and PT2 cells generated higher levels of global ROS compared to two different lots of normal astrocytes. PT4 and PT2 cells also displayed a tight regulation of the endogenous ROS levels, even in the presence of NAC and tiron. Trolox was slightly more potent in this regard, since it indeed showed the ability to reduce global ROS levels. Notably, the three antioxidants used in this study did not modify the mitochondrial proton gradient.

We showed that alterations in the phosphorylation pattern of three major proteins involved in the response to changes of redox status, could not explain in a straightforward manner the cell survival decrease caused by NAC, tiron and trolox, as reported for NAC in a different experimental context [19].

Concerning cell survival, our data showed that the three drugs used in the study caused a dose-dependent inhibition of cell growth and that apoptosis had only a minor role in this. We observed that NAC and tiron increased the percentage of cells in the G0/G1 and S phase of the cell cycle, respectively, in both cell cultures containing GBM TICs (PT2 and PT4). The observed prolongation of the G1 phase induced by NAC was previously reported [48] although several differences were noted here. For example we showed that the increased fraction of G0/G1 cells occurred also with trolox in PT2 cells. Moreover, unlike the previous study [48], we neither observed induction of CDKN1A/P21WAF by NAC nor of the *CDKN2A* transcripts in PT4 cells. This last result was expected since these cells displayed a biallelic loss of the *CDKN2A*. Lastly, both our study and the previous one [48] determined in good agreement that the antioxidant properties of NAC were not involved in the G0/G1 increase.

We also ruled out that a reduced proliferation was the result of an induction of a possible differentiation of the cells in the culture containing GBM TICs caused by the antioxidants. Indeed we showed no major changes in the expression of stem cell markers such as Nestin, SOX2 and OLIG2 in treated cells after 6 days of exposure and no induction of several other differentiation markers.

The gene expression profiles of cultures containing GBM TICs following the treatment with the NAC, tiron and trolox showed that these drugs impaired cell survival by different mechanisms. First, NAC, tiron and trolox modulated only a negligible shared number of genes. Second, we also found that the timing of gene modulation was different. In fact, while both NAC and trolox treatments modulated more genes at 48 h than at 6 days, the opposite was shown for tiron. Last but not least, the analysis of gene expression profiles indicated that NAC and trolox impaired cell survival by inhibiting cell cycle progression at the earliest treatment time-point. Tiron, instead, first induced a hypoxia-like condition which likely caused inhibition of the cell cycle progression at a later time. These data were in agreement with those of a previous report which showed that tiron induced hypoxia response elements and HIF1alpha expression under normoxic conditions [49]. Our data also suggested that TP53 and RB might mediate the inhibition of cell cycle progression displayed by NAC, tiron and trolox, along with CDKN1A/P21WAF in tiron treated PT4 cells. These findings are also in agreement with a previous report showing posttranscriptional regulation of P53 levels via enhanced translation of the TP53 transcript by NAC [50]. However, while this previous study reported a TP53 dependent apoptosis, induction of apoptosis was negligible in our context. Our data concerning RB hypophosphorylation induced by NAC are also in agreement with a previous report on colorectal carcinoma cells [51]. Instead, to the best of our knowledge, the hypophosphorylation of RB induced by tiron and trolox is a novel finding.

In this study we also observed that the exposure of both PT4 and PT2 cell cultures to tiron induced an increased fraction of cells to enter the S phase along with a reduced percentage of BrdU positive cells. This data can be explained with an increase of S0 (or S quiescent cells). Several studies have shown that it is possible to detect cells within the S phase compartment (cells with a DNA content intermediate between those of G0/G1, and G2/M cells) that are not actively replicating their DNA [52]–[59]. Both the increased fraction of cells in S phase and the increase of S quiescent cells caused by tiron might be explained with the induction of a hypoxia-like status. In support of this hypothesis, previous reports showed an increase of the S phase fraction of hypoxic mesenchymal stem cells compared to normoxic controls [60] and the existence of a correlation between the fraction of quiescent S-phase cells and the degree of hypoxia in xenografted human tumors and colon cancers cells in vitro [56], [59]. Tiron was reported to induce HIF-1alpha, in accordance with our findings at 6 days of treatment, while some biological effects of this drug, including the observed cytokinesis-block, were hypothesized to result from disturbance of iron metabolism [61]. Concerning the regulation of hypoxia related genes observed at 6 days of treatment with NAC, it should be noted that only two of the seven genes belonging to this category were upregulated. Instead, all sixteen genes included in the response to oxygen levels were upregulated after 48 hours of tiron treatment as expected in a hypoxia status. Therefore we concluded that tiron but not NAC has the potential to cause a hypoxia-like condition in our system. Interestingly, tiron also induced all the genes associated with the angiogenesis GO biological process, including *VEGFA*. In our opinion this relationship suggests that tiron could actually promote tumor growth and metastasis *in vivo*.

The analysis of the gene expression profiles showed that only trolox promoted a deregulation of genes related to redox biological processes. However, the statistically significant P-value associated with the GO oxidation reduction biological process at 6 days of treatment was four orders of magnitude less than the third top ranking process at the same time point (data not shown).

Our data also showed that 6 days of treatment with tiron or trolox inhibited survival of cells cultures containing TICs from a malignant mesothelioma and a melanoma. This demonstrated that the cell survival impairment displayed by these drugs was not specific to cultures containing GBM TICs or TICs established from a specific individual. However, results obtained after longer exposure of FO-1 cells to trolox suggested that these cells may develop a resistance mechanism toward the growth-inhibition effect of this drug.

Concerning NAC, our results suggested that its inhibitory effect on cell survival could be inversely related to the degree of activation of the EGFR/RAS/BRAF pathway. On the other hand our data also showed that NAC, trolox and tiron enhanced the effects of inhibitors of two kinases belonging to this pathway, although to various extents.

Collectively, we showed that NAC, tiron and trolox acted as wide modulators of gene expression and that they impaired cell survival by inhibiting cell cycle progression. In our experimental context their biological activities were not related to the modulation of cell redox balance.

## Supporting Information

Figure S1 **Cell cultures containing GBM TICs display higher endogenous global ROS generation than normal astrocytes.** Representative experiment of FCM analysis of normal human astrocytes (NA2) and cell cultures containing GBM TICs obtained from two different patients (PT2 and PT4) incubated with the indicated fluorescent probe. Mitotracker Green, TMRE, DCFDA and MitoSOX Red were used to evaluate mitochondrial mass, mitochondrial proton gradient, global ROS and mitochondrial superoxide, respectively.
(TIF)

Figure S2 **Phase contrast images of PT4 cell culture containing TICs treated for 6 days with the indicated substances at the respective IC50 doses.** Bar = 100 micrometers.
(TIF)

Figure S3 **Cell survival of the PT4, MM1 and FO-1 cell cultures containing TICs.** Survival of PT4 cells (A, E); MM1 cells (B); FO-1 cells (C, D) after 6 days of treatment with the indicated substances and solvent controls. The medium renewal schedule was identical to that used for the cultures containing GBM TICs (see Introduction). Cell survival is expressed in arbitrary units as evaluated by MTT analysis. Standard deviations are indicated as vertical bars (n = 3 independent assays). DMSO concentration in (D) was 0.1% vol/voI. Drug concentrations in (D) were: NAC 20 mM, PLX4032 10 µM. Gefitinib final concentration in (E) was 3.9 µM. #The unpaired t-test was significant at P<0.05. §The unpaired t-test was significant at P = 0.01 or less. *The unpaired t-test was significant at P<0.001. **The unpaired t-test was significant at P<0.0001.
(TIF)

Figure S4 **Survival of PT2 cells after 6 days of treatment with the indicated substances and solvent controls.** The medium renewal schedule was identical to that used far the cultures containing GBM TICs (see Introduction). Cell survival is expressed in arbitrary units as evaluated by MTT analysis. Standard deviations are indicated as vertical bars (n = 4 independent assays). §The unpaired t-test was significant at P<0.01. *The unpaired t-test was significant at P<0.001.
(TIF)

Figure S5 **NAC, tiron and trolox modify the distribution in the cell cycle phases of the PT2 cell culture containing GBM TICs.** Representative experiment of high resolution FCM analysis of DAPI-stained nuclei of the H_2_O control PT4 culture containing GBM TICs after 48 h of exposure (A). Analysis of the percentage of PT4 cells in the cell cycle phases as determined by the ModFit LT™ software after 48 h of exposure with the indicated substances at the IC50 concentration and solvent controls (B). This analysis revealed with respect to solvent controls: a higher percentage of cells in the G0/G1 phase when treated with NAC (with concomitant reduction of cells in the G2/M phase) and a higher percentage of cells in the S phase when treated with tiron. Standard deviations are indicated as vertical bars (n = 5 independent assays, B; n = 3 independent assays, C). §The unpaired t-test was significant at P = 0.01 or less. *The unpaired t-test was significant at P<0.001. **The unpaired t-test was significant at P<0.0001. ***The unpaired t-test was significant at P<0.00001.
(TIF)

Figure S6 **NAC and tiron cause only modest changes in ROS levels in the PT2 cell culture containing GBM TICs, whereas trolox decreases global ROS but not mitochondrial ROS levels.** Representative experiment of FCM analysis of PT2 culture containing GBM TICs cells incubated with the indicated fluorescent probe after 48 h of exposure with the indicated substances and solvent controls. The experiment was performed immediately after a fresh media (containing NAC, tiron or trolox) replacement. DCFDA, MitoSOX Red and TMRE were used to evaluate global ROS, mitochondrial superoxide and mitochondrial proton gradient, respectively. This analysis showed that trolox reduced global cellular ROS levels but slightly enhanced mitochondrial superoxide levels. NAC and tiron, instead, while slightly decreased mitochondrial superoxide levels, slightly enhanced global cellular ROS levels. This analysis also showed that the drugs used in this study induced no changes of the mitochondrial proton gradient displayed by the PT2 cells in control conditions.
(TIF)

Figure S7 **Phosphorylation status of AKT, ERK1/2 and NF-kB in the PT4 cell culture containing GBM TlCs resulting from a typical experiment of 48 h exposure to the indicated substances.** The figure shows immunoblot analysis of cell Iysates with specific antibodies able to detect either specific phosphorylated isoforms of the indicated proteins or the same proteins independently from the phosphorylation status (see Text S1 for details). Each immunoblotted membrane was subjected to multiple antibody challenging, stripping, control of effective stripping, and rechallenging with a different antibody. The last antibody used was an anti tubulin alpha to show equal loading. The immunoblot image did not contain saturated pixels.
(TIF)

Figure S8 **Global comparison among probe sets found deregulated in PT4 cell culture containing GBM TICs by NAC, trolox and tiron with respect to solvent controls at 48 h (A) and 6 days (B) of treatment, represented as Venn diagrams.** The number of probe sets associated to coregulated genes is reported in the overlapping areas.
(TIF)

Figure S9 **Western blot analyses performed with Iysates of the PT4 cell culture containing GBM TICs treated for 6 days with the indicated antioxidant drug and challenged with anti MKi67, Pdz-binding kinase (PBK), transferrin receptor (TFRC), carbonic anhydrase 9 (CA9) antibodies.** The immunoblotted membrane was subjected to multiple antibody challenging, stripping, control of effective stripping, and rechallenging with a different antibody. The last antibody used was an anti histone deacetylase 1 (HDAC1) to show equal loading.
(TIF)

Table S1 **Sequences accession numbers and primers used for qPCR analysis.**
(XLSX)

Table S2 **List of genes deregulated by NAC, tiron and trolox vs. solvent controls, after 48 h and 6 days of treatment, included in the three top ranking GO biological processes as determined by DAVID tools (see MM).**
(XLSX)

Table S3 **Expression values for probe sets related to genes coding for nervous system markers.** Gray fill indicates probe sets with expression levels below the 250 arbitrary cut off chose for the analysis of fold regulation.
(XLSX)

Text S1 **Antibodies used in the study.**
(DOC)
