# Supplementary figures and images for: NAC, Tiron and Trolox Impair Survival of Cell Cultures Containing Glioblastoma Tumorigenic Initiating Cells by Inhibition of Cell Cycle Progression
Source: PLoS One. 2014 Feb 28;9(2):e90085. doi: 10.1371/journal.pone.0090085 (PMC3938592; doi:10.1371/journal.pone.0090085)

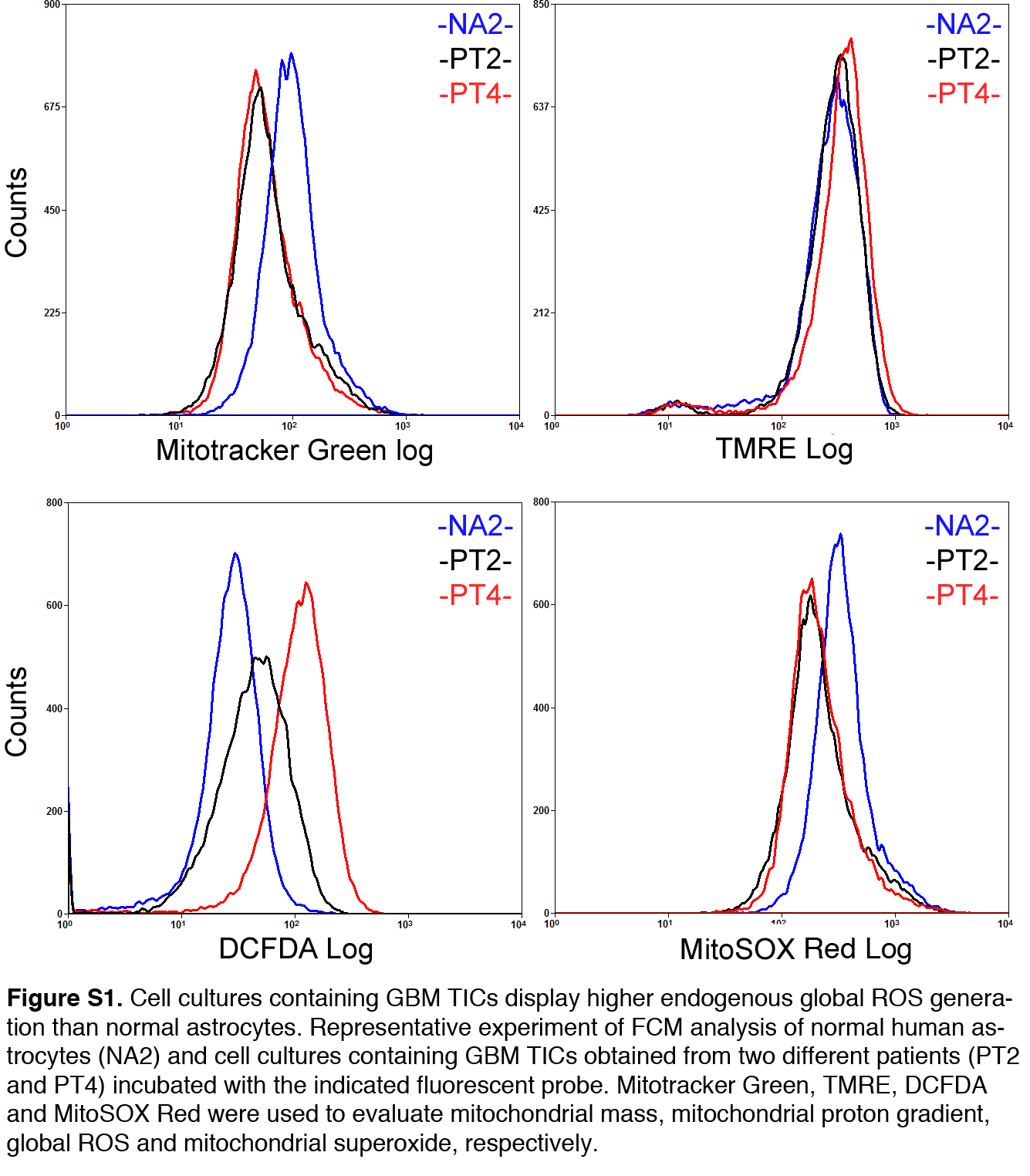

Supplement: Figure S1 — Cell cultures containing GBM TICs display higher endogenous global ROS generation than normal astrocytes. Representative experiment of FCM analysis of normal human astrocytes (NA2) and cell cultures containing GBM TICs obtained from two different patients (PT2 and PT4) incubated with the indicated fluorescent probe. Mitotracker Green, TMRE, DCFDA and MitoSOX Red were used to evaluate mitochondrial mass, mitochondrial proton gradient, global ROS and mitochondrial superoxide, respectively. (TIF) [file pone.0090085.s001.tif]

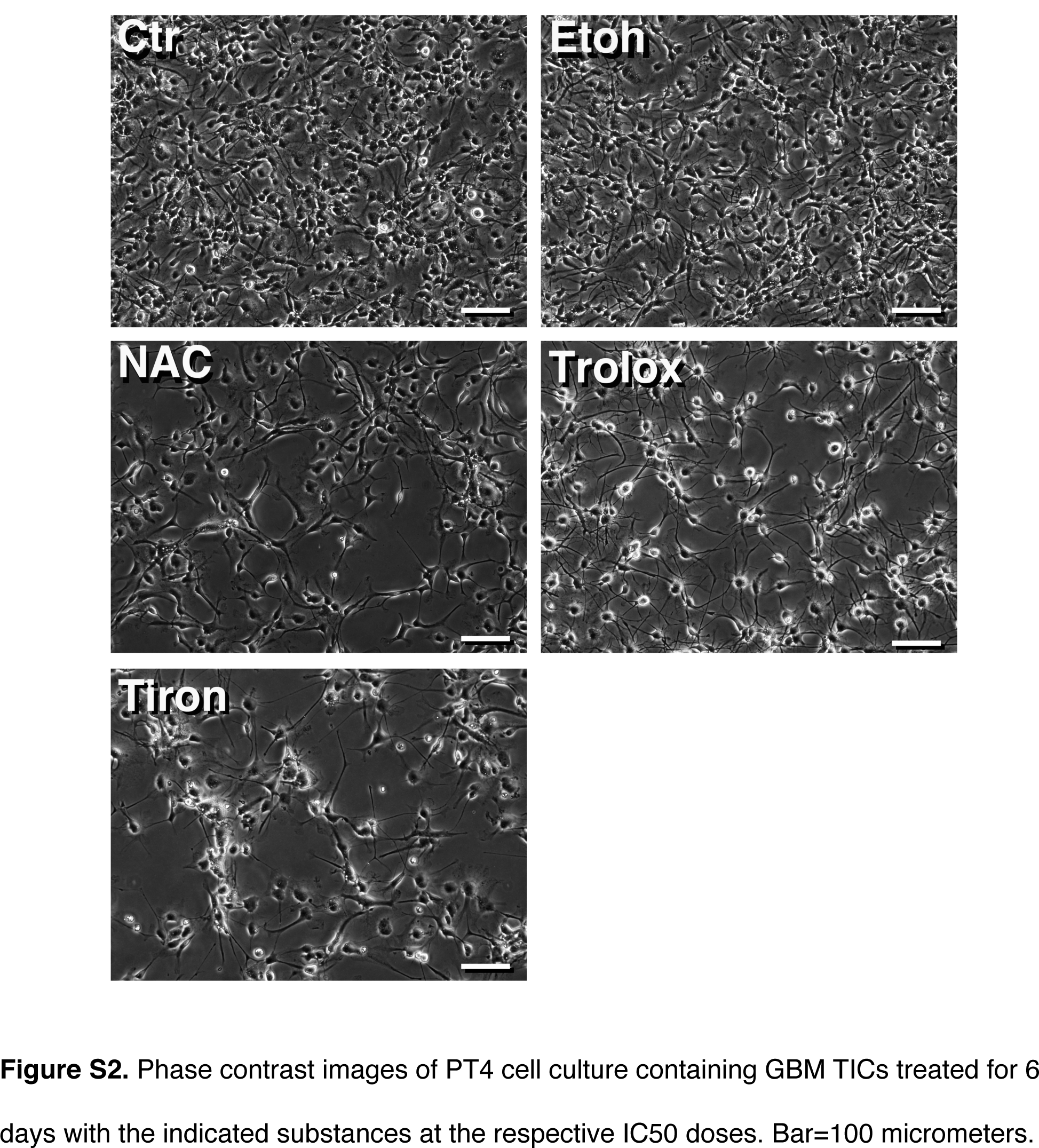

Supplement: Figure S2 — Phase contrast images of PT4 cell culture containing TICs treated for 6 days with the indicated substances at the respective IC50 doses. Bar = 100 micrometers. (TIF) [file pone.0090085.s002.tif]

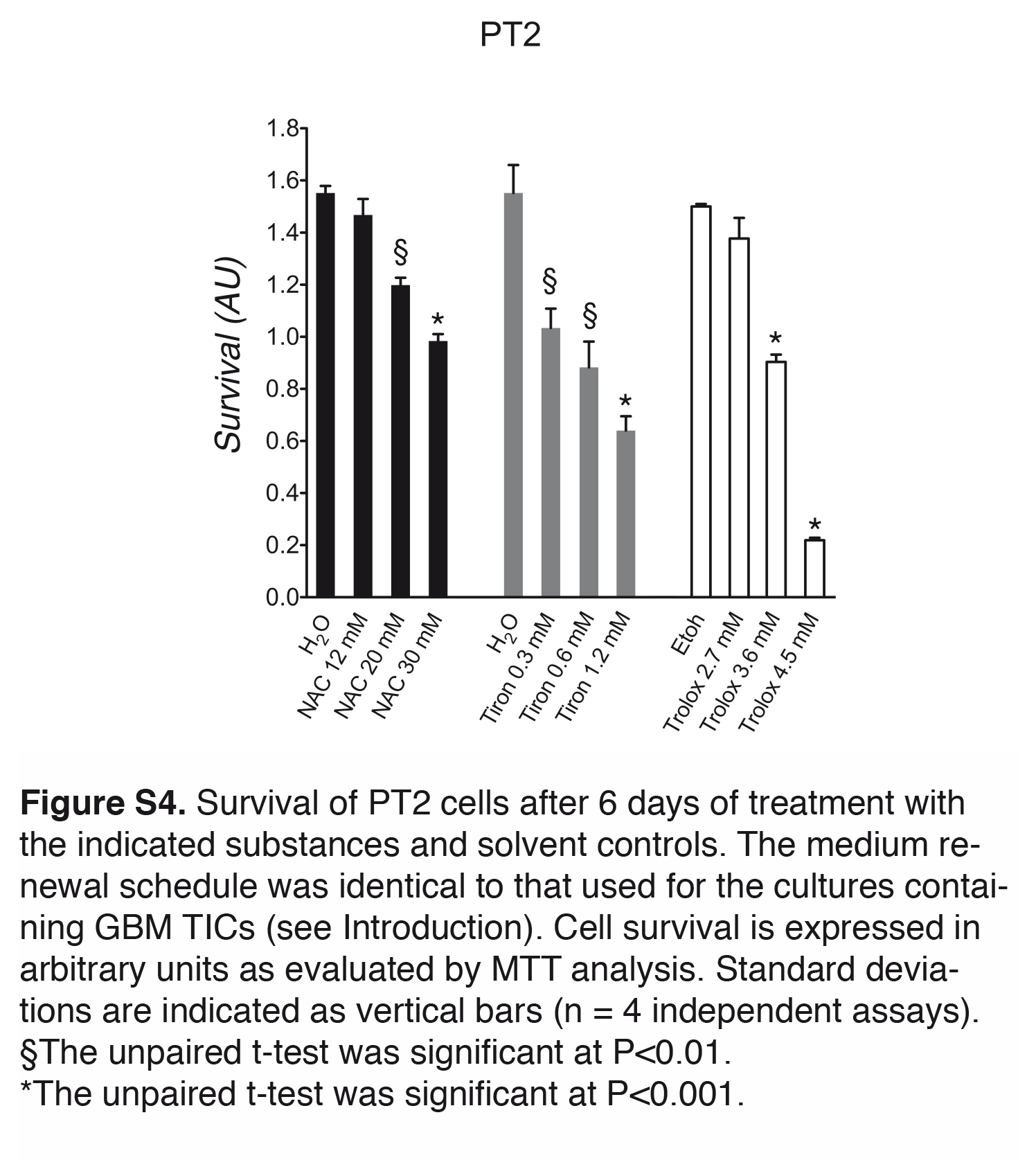

Supplement: Figure S4 — Survival of PT2 cells after 6 days of treatment with the indicated substances and solvent controls. The medium renewal schedule was identical to that used far the cultures containing GBM TICs (see Introduction). Cell survival is expressed in arbitrary units as evaluated by MTT analysis. Standard deviations are indicated as vertical bars (n = 4 independent assays). §The unpaired t-test was significant at P<0.01. *The unpaired t-test was significant at P<0.001. (TIF) [file pone.0090085.s004.tif]

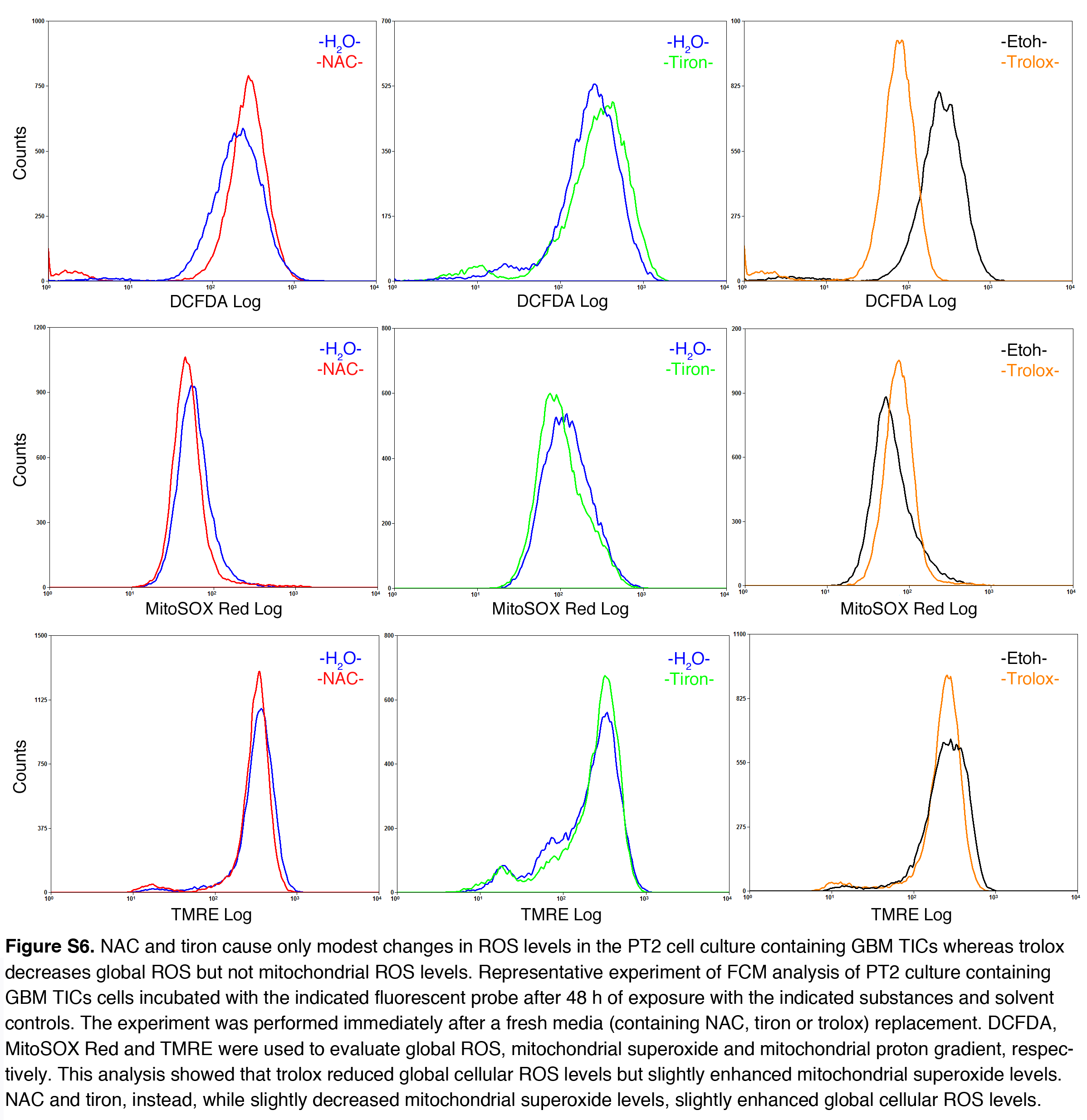

Supplement: Figure S6 — NAC and tiron cause only modest changes in ROS levels in the PT2 cell culture containing GBM TICs, whereas trolox decreases global ROS but not mitochondrial ROS levels. Representative experiment of FCM analysis of PT2 culture containing GBM TICs cells incubated with the indicated fluorescent probe after 48 h of exposure with the indicated substances and solvent controls. The experiment was performed immediately after a fresh media (containing NAC, tiron or trolox) replacement. DCFDA, MitoSOX Red and TMRE were used to evaluate global ROS, mitochondrial superoxide and mitochondrial proton gradient, respectively. This analysis showed that trolox reduced global cellular ROS levels but slightly enhanced mitochondrial superoxide levels. NAC and tiron, instead, while slightly decreased mitochondrial superoxide levels, slightly enhanced global cellular ROS levels. This analysis also showed that the drugs used in this study induced no changes of the mitochondrial proton gradient displayed by the PT2 cells in control conditions. (TIF) [file pone.0090085.s006.tif]

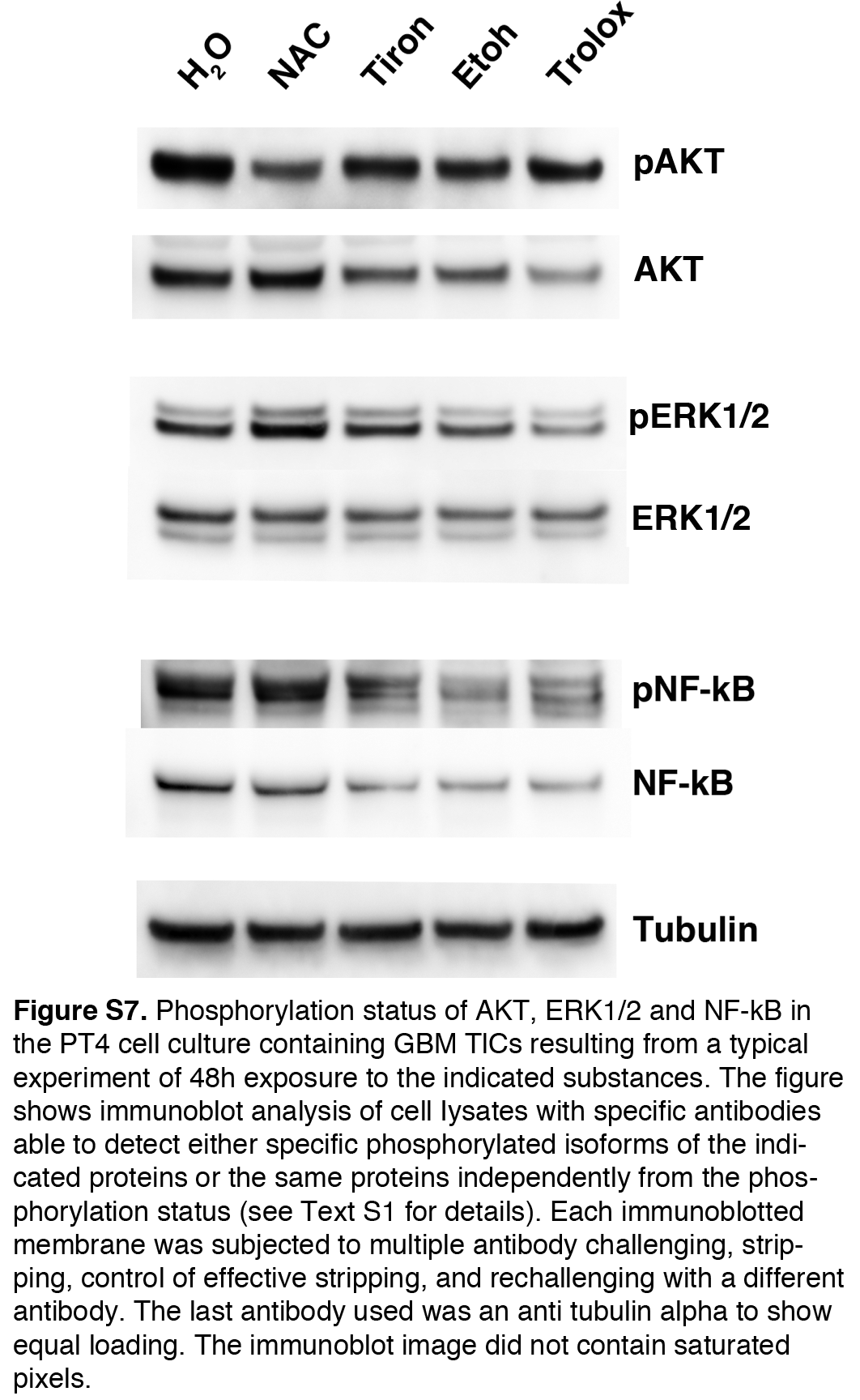

Supplement: Figure S7 — Phosphorylation status of AKT, ERK1/2 and NF-kB in the PT4 cell culture containing GBM TlCs resulting from a typical experiment of 48 h exposure to the indicated substances. The figure shows immunoblot analysis of cell Iysates with specific antibodies able to detect either specific phosphorylated isoforms of the indicated proteins or the same proteins independently from the phosphorylation status (see Text S1 for details). Each immunoblotted membrane was subjected to multiple antibody challenging, stripping, control of effective stripping, and rechallenging with a different antibody. The last antibody used was an anti tubulin alpha to show equal loading. The immunoblot image did not contain saturated pixels. (TIF) [file pone.0090085.s007.tif]

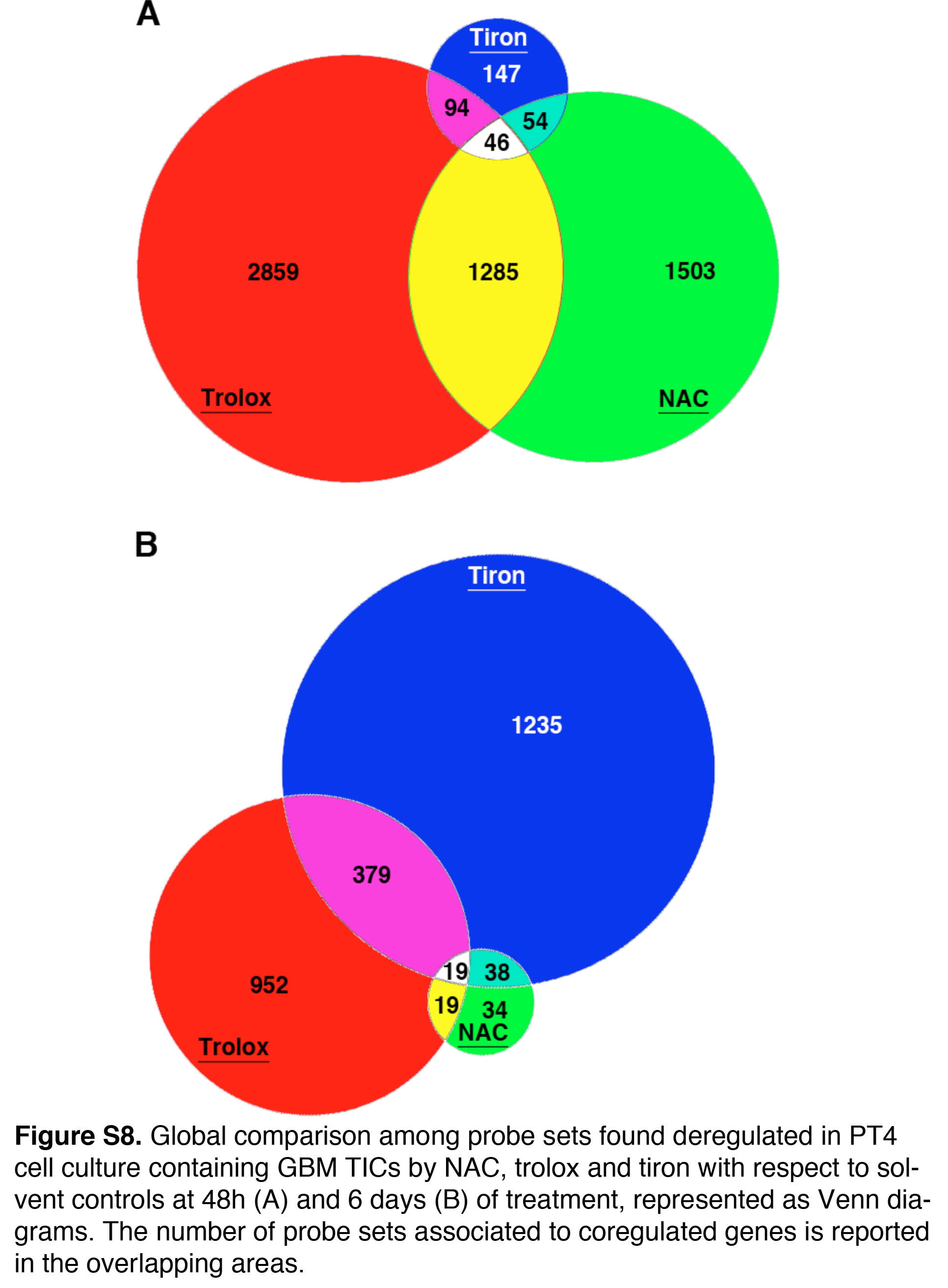

Supplement: Figure S8 — Global comparison among probe sets found deregulated in PT4 cell culture containing GBM TICs by NAC, trolox and tiron with respect to solvent controls at 48 h (A) and 6 days (B) of treatment, represented as Venn diagrams. The number of probe sets associated to coregulated genes is reported in the overlapping areas. (TIF) [file pone.0090085.s008.tif]

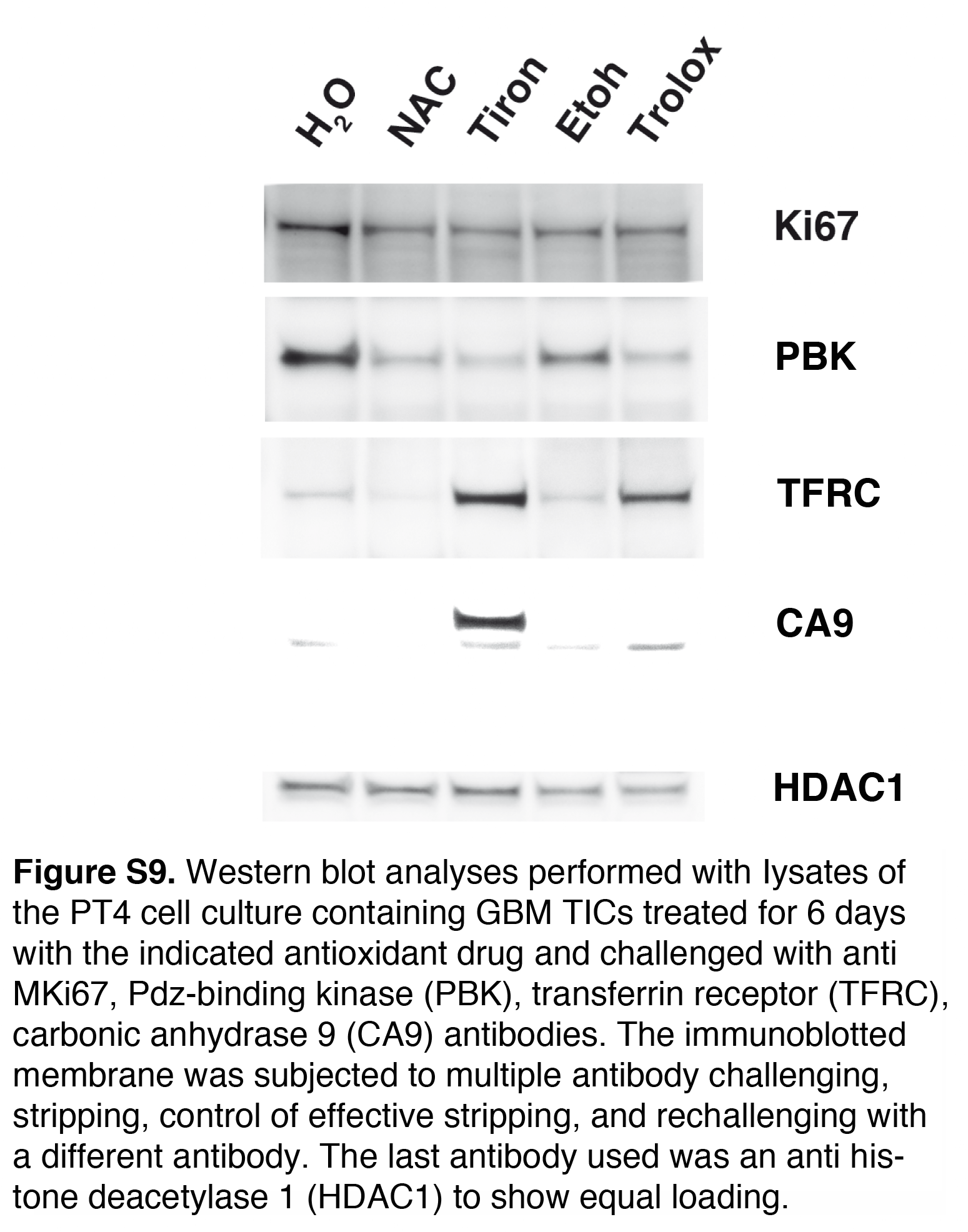

Supplement: Figure S9 — Western blot analyses performed with Iysates of the PT4 cell culture containing GBM TICs treated for 6 days with the indicated antioxidant drug and challenged with anti MKi67, Pdz-binding kinase (PBK), transferrin receptor (TFRC), carbonic anhydrase 9 (CA9) antibodies. The immunoblotted membrane was subjected to multiple antibody challenging, stripping, control of effective stripping, and rechallenging with a different antibody. The last antibody used was an anti histone deacetylase 1 (HDAC1) to show equal loading. (TIF) [file pone.0090085.s009.tif]
