# Supplementary material for: NAC, Tiron and Trolox Impair Survival of Cell Cultures Containing Glioblastoma Tumorigenic Initiating Cells by Inhibition of Cell Cycle Progression
Source: PLoS One. 2014 Feb 28;9(2):e90085. doi: 10.1371/journal.pone.0090085 (PMC3938592; doi:10.1371/journal.pone.0090085)
Supplement: Text S1 — Antibodies used in the study. (DOC) [file pone.0090085.s013.doc]

Text S1. Antibodies used in the study.

The mAb M12 anti PDZ-Binding Kinase (PBK) was purchased from Abnova (Taipei, Taiwan) and the mAb M75 anti Carbonic Anhydrase IX (CA9) from Bioscience (Bratislava, Slovakia).

The anti cyclin-dependent kinase inhibitor 1A (CDKN1A/P21^waf^) antibody (C19) were purchased from Santa Cruz Biotechnology (Santa Cruz Biotechnology, Santa Cruz, CA, USA) and the anti SOX2 from Millipore.

The antibodies anti phospho-AKT (Ser473), AKT, phospho-ERK1/2 (Thr202/Tyr204), ERK1/2, phospho Rb (Ser 807/811), P53 and anti total Rb were purchased from Cell Signaling Technology Inc. (Danvers, MA, USA).

The antibodies anti phospho-NF-kB (S276)(ab30623), transferrin receptor (TFRC) (ab84036), and Ki67 (ab15580) were purchased from Abcam (Cambridge, UK).

To show equal loading of the protein gels we used the detection of either alpha tubulin or histone deacetylase 1, since the transcript for both proteins did not show major variation in abundances across the experimental conditions and solvent controls (data not shown).

The anti Histone Deacetylase 1 (HDAC1) antibody (H3284) and the anti alpha tubulin (Clone B-5-1-2) were from Sigma-Aldrich.
